# Increased levels of histidine-rich glycoprotein are associated with the development of post-thrombotic syndrome

**DOI:** 10.1038/s41598-020-71437-5

**Published:** 2020-09-02

**Authors:** Jakub Siudut, Joanna Natorska, Maksim Son, Krzysztof Plens, Anetta Undas

**Affiliations:** 1 Krakow Centre for Medical Research and Technologies, John Paul II Hospital, Krakow, Poland; 2 Institute of Cardiology, Jagiellonian University Medical College, 80 Pradnicka St, 31-202 Krakow, Poland; 3 Department of Clinical Neurological Sciences, University of Western Ontario, London, Canada; 4 KCRI, Krakow, Poland

**Keywords:** Predictive markers, Thromboembolism

## Abstract

Denser fibrin networks which are relatively resistant to lysis can predispose to post-thrombotic syndrome (PTS). Histidine-rich glycoprotein (HRG), a blood protein displaying antifibrinolytic properties, is present in fibrin clots. We investigated whether HRG may affect the risk of PTS in relation to alterations to fibrin characteristics. In venous thromboembolism (VTE) patients, we evaluated plasma HRG levels, plasma clot permeability, maximum absorbance, clot lysis time and maximum rate of increase in D-dimer levels released from clots after 3 months of the index event. We excluded patients with cancer and severe comorbidities. After 2 years of follow-up, 48 patients who developed PTS had 18.6% higher HRG at baseline. Baseline HRG positively correlated with clot lysis time, maximum absorbance, and thrombin-activatable fibrinolysis inhibitor (TAFI) activity but was inversely correlated with plasma clot permeability and maximum rate of increase in D-dimer levels released from clots. On multivariate regression model adjusted for age, fibrinogen and glucose, independent predictors of PTS were recurrent VTE, baseline HRG level, and TAFI activity. VTE recurred in 45 patients, including 30 patients with PTS, and this event showed no association with elevated HRG. Our findings suggest that increased HRG levels might contribute to the development of PTS, in part through prothrombotic fibrin clot properties.

## Introduction

Post-thrombotic syndrome (PTS) affects as many as 23–60% of patients in the first 2 years of deep-vein thrombosis (DVT)^1^. It is a constellation of signs and symptoms of chronic deep venous insufficiency and typically manifests as swelling, pain, peripheral oedema, venous ectasia, and in advanced cases—ulceration. The severity is usually determined using the Villalta scale^2^.

PTS is thought to arise from chronic thrombotic obstruction of the deep veins leading to venous hypertension, exacerbated by valvular incompetence^3^. Residual vein obstruction post DVT has been reported to be linked to impaired fibrinolysis and disturbed microcirculation^4–7^. The exact mechanism underlying PTS is poorly understood. PTS results at least in part from delayed venous thrombus resolution and induction of vein wall fibrosis, which promotes valvular reflux^8^. Robust evidence indicates that PTS is closely associated with enhanced systemic inflammation^3^.

Fibrin formation is the final stage of blood coagulation. Fibrin clot structure is highly heterogeneous and determined by several genetic and environmental factors, with a commonly observed prothrombotic phenotype involving the formation of denser fibrin networks which are relatively resistant to lysis^9^. Such altered fibrin clot properties have been observed in unprovoked venous thromboembolism (VTE)^9^. The prothrombotic clot phenotype has also been reported to increase risk of recurrent DVT^10^. In 2016 Siudut et al. found that lowered fibrin clot permeability and impaired lysis assessed off anticoagulation following a few months since the first DVT predispose patients to develop PTS^11^.

Histidine-rich glycoprotein (HRG), an abundant plasma protein synthesized by the liver, has a host of properties including anti-inflammatory effects, along with both anticoagulant and antifibrinolytic activity^12,13^. HRG levels decrease during sepsis^13^, as well as in patients with advanced cancer^14,15^. The role of HRG in blood coagulation in humans remains unclear. HRG is known to bind heparan sulfate, tropomyosin, and heme^16^. HRG may limit antithrombin activity by binding heparin at the N-terminal heparin binding site, due to a high degree of sequence homology between HRG and antithrombin^17^. In addition, in vitro studies showed that HRG effectively binds 50% of circulating plasminogen^16^. It is unclear whether HRG has any effect on the conversion of fibrinogen to fibrin, however its incorporation into fibrin clots has been shown to lead to formation of thinner fibrin fibres in vitro^18^. Mice that lacked HRG expression had higher spontaneous fibrinolytic activity, but also shorter prothrombin time and bleeding time^19^. Of note, recent proteomic analyses have confirmed the presence of HRG in human plasma clots in healthy subjects and VTE patients^20^.

Data on the association of HRG with VTE are conflicting. Some studies have reported elevated HRG in patients with VTE^21,22^, while others found that HRG deficiency is a risk factor for VTE^23,24^. To our knowledge, there have been no reports exploring HRG in patients with PTS. Based on available data, we hypothesized that elevated HRG may contribute to long-term sequelae of DVT in part through prothrombotic alterations to fibrin characteristics.

## Patients and methods

### Baseline characteristics

We screened 243 Caucasian patients aged between 18 to 70 years with a history of first-ever DVT between October 2008 and June 2010. The patients represented a subgroup of the original cohort of patients with VTE described previously^11^. Of the 243 individuals with DVT, we excluded 46 patients. The exclusion criteria were: deficiency of antithrombin, protein C or protein S, antiphospholipid syndrome, acute coronary syndrome or ischaemic stroke within the previous 3 months, known malignancy, any inflammatory states (C-reactive protein [CRP] > 15 mg/L), diabetes, advanced chronic renal disease, international normalized ratio (INR) more than 1.2, all the states reportedly associated with abnormal plasma clot properties^9^. Furthermore, we excluded 15 patients in whom baseline plasma samples to measure HRG levels were not available. Overall, 182 individuals were included in our final analysis.

DVT was diagnosed with duplex sonography (presence of an intraluminal thrombus in the calf, popliteal, femoral or iliac veins) and proximal DVT was diagnosed if the thrombus was detected in the popliteal veins (including trifurcation), femoral, and iliac veins. The diagnosis of PE was based on the clinical history and computed tomography (CT) angiography. All patients initially were treated with unfractionated or low-molecular-weight heparins and then with vitamin K antagonists (VKA). Duration of anticoagulation was 3 months or more (in patients with provoked VTE), or longer (for the patients with unprovoked VTE and/or recurrence of VTE) at the discretion of treating physicians. VTE was classified as unprovoked if there was no history of cancer, surgery requiring general anesthesia, major trauma, plaster cast or hospitalization within the last month, or pregnancy/delivery within the last 3 months. Class II custom-fitted elastic compression stockings or hosiery were prescribed to patients for a period of 6–24 months.

The research has been compliant with all relevant national and institutional regulations. Experimental protocols were approved by Jagiellonian University Ethical Committee and all patients gave the informed consent in accordance with the Declaration of Helsinki.

### Follow-up

**Figure 1:**
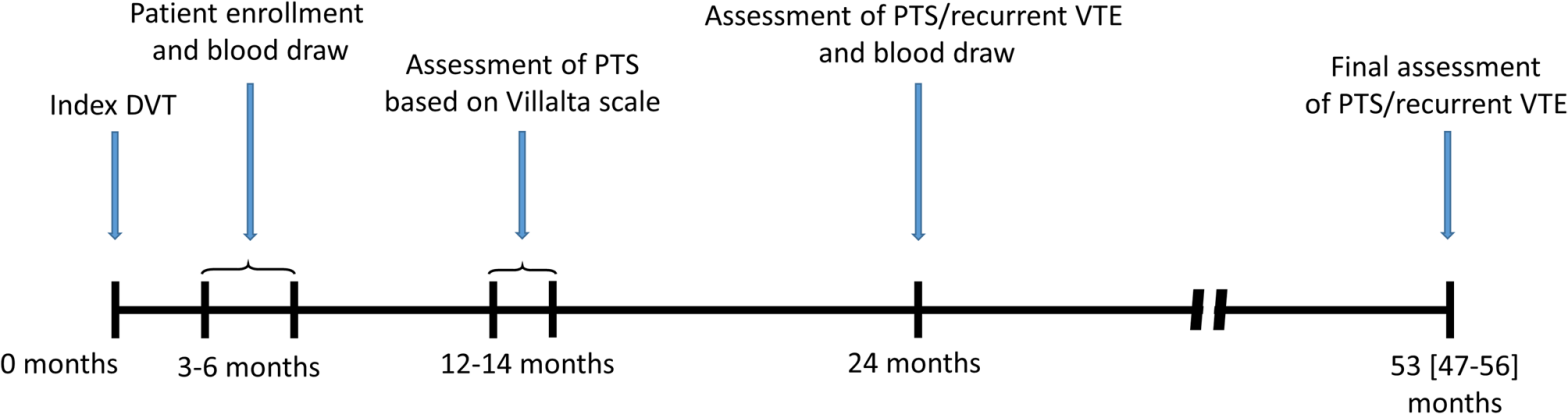
A flow-chart representing patient enrollment and long-term observation.

PTS was assessed 12–14 months since the index event using the Villalta scale^2^ and was defined as a score of ≥ 5 on two consecutive visits that were at least 3 months apart. After 24 months all patients had prespecified assessment of PTS at the clinic. We evaluated symptomatic recurrent DVT or symptomatic PE. In subjects with signs or symptoms suggestive of recurrent DVT, including enhanced pain, or tenderness, oedema, and redness, if there was incompressibility of a proximal vein segment previously free from thrombi, or the finding of a more than 4 mm increase of the vein diameter in a previously non-compressible vein segment as compared with the last available measurement. Patients suspected for PE underwent spiral computed tomography, followed by pulmonary angiography in the case of a high clinical probability of PE despite normal CT scans. After a median of 53 (IQR 47–56) months patients underwent the final assessment for PTS and symptomatic recurrent VTE (Fig. 1).

### Laboratory investigations

Blood samples were drawn from an antecubital vein with minimal stasis at 08:00–10:00 off anticoagulation 3–6 months and 24 months since the index event. Patients on VKA were switched to anticoagulation with low-molecular-weight heparin for 10–14 days and blood was collected ≥ 12 h after the last injection when the INR was less than 1.2. Venous blood samples were centrifuged at 2000* g* for 10 min within 30 min of the draw, and the supernatant was aliquoted and stored at − 80 °C until analysis. Routine laboratory tests were used to evaluate lipid profile, glucose, and creatinine. Fibrinogen was assessed using the Clauss assay. High-sensitivity CRP was measured by immunoturbidimetry (Roche Diagnostics GmbH, Mannheim, Germany). Plasma D-dimer was measured with the Innovance D-dimer assay (Siemens, Marburg, Germany). We measured HRG antigen (Cusabio Biotech Co., Ltd., Wuhan, China) according to the manufacturer’s instructions at baseline and 2 years after the initial HRG measurement. Tissue-type plasminogen activator (tPA), plasminogen activator inhibitor-1 (PAI-1) antigens, and plasma-activated thrombin activatable fibrinolysis inhibitor (TAFI) activity (all American Diagnostica, Stamford, CT, USA), together with interleukin (IL)-6 and IL-10 (both Quantikine, R&D Systems, Inc., Minneapolis, MN, Canada) levels were measured using ELISA kits. Plasminogen and α-antiplasmin activities were measured using chromogenic assays (both Diagnostica Stago, Asnières, France). Inter- and intra-assay variation coefficients were < 8%. Plasma Factor VIII activity was determined using a one-stage clotting assay (Siemens). Thrombophilia screening was performed in all patients as previously described^10^.

### Fibrin clot permeability

Fibrin clot permeability was determined using a pressure-driven system^25^. Briefly, 20 mmol/L CaCl_2_ and 1 U/mL human thrombin (Sigma, St Louis, MO, USA) were added to 120 µL citrated plasma. After 2 h of incubation in a wet chamber, buffer volume flowing through the gels was measured. Plasma clot permeability indicating the average pore size within the clot, was calculated using the formula Q × L × µ/t × A × Δp, where Q is the flow rate; L, length of the fibrin gel; µ, viscosity of the liquid (in poise); A, a cross-sectional area (in cm^2^), Δp, a differential pressure (in dyne cm^2^) and t, time.

### Clot lysis analysis

Two different methods were used to assess the efficiency of clot lysis. In the first assay^26^, citrated plasma was mixed with 15 mmol/L CaCl_2_, 0.6 pM human tissue factor (Innovin, Siemens), 12 µmol/L phospholipid vesicles and 60 ng/mL recombinant tPA (Boehringer Ingelheim, Ingelheim, Germany). The turbidity was measured at 405 nm at 37 °C. The lag phase of the turbidity curve, which reflects the time required for initial protofibril formation and maximum absorbance at the plateau phase were assessed^11^. Clot lysis time was defined as the time from the midpoint of the clear-to-maximum-turbid transition to the mid-point of the maximum-turbid-to-clear transition. In the second assay, fibrin clots, formed as above, were perfused with a Tris buffer containing 0.2 µmol/L rtPA (Boehringer Ingelheim). D-dimer levels were measured every 20 min in the effluent using an ELISA (American Diagnostica) for 120 min and the maximum rate of increase in D-dimer levels and maximum levels of D‐dimer released from clots were analysed. All measurements were performed by technicians blinded to the origin of the samples. Inter- and intra-assay variation coefficients of the measurements were 6–9%.

### Statistical analysis

Continuous variables were expressed as mean and standard deviation (SD) or median and interquartile range (IQR) as appropriate. Categorical variables were presented as numbers and percentages and compared by the Fisher’s exact test. Normality was assessed by the Shapiro–Wilk test. Equality of variances was assessed using the Levene’s test. Differences between the groups were compared using the Student’s or the Welch’s t-test depending on the equality of variances for normally distributed variables. The Mann–Whitney U-test was used for non-normally distributed variables. The Spearman’s rank correlation coefficient or the Pearson’s correlation coefficient were used to measure the linear associations between two variables. The Benjamini–Hochberg procedure was used to control false discovery rate for multiple comparisons. The risk of PTS was determined by univariate and multivariate models. Variable selection in the multivariate models were performed using least absolute shrinkage and selection operator (LASSO) using tenfold cross validation to obtain optimal values for λ^27^. Two-sided *p* -values < 0.05 were considered statistically significant. All calculations were done with JMP, Version 9.0.0 (SAS Institute Inc., Cary, NC, USA, 1989–2007).

## Results

We included 182 patients at a mean age of 45 years (range 18 to 69 years) to the final analysis (Table 1). The group comprised 102 men (56%) and 80 women (44%). Fifty-five patients (30.2%) had symptomatic PE combined with DVT, while the majority experienced DVT alone.

**Table 1:** Baseline patient characteristics.

Variable	Total cohort (n = 182)	PTS (n = 48)	Non-PTS (n = 134)	*p*-value
Age, years	45 (33–55)	49 (41–58)	43 (30–52)	0.022
Male *n,* (%)	102 (56)	26 (54.2)	76 (56.7)	0.577
BMI, kg/m^2^	26.5 (24.8–29.2)	27.3 (25.1–30.1)	26.2 (24.5–28.5)	0.029
**Blood type, n (%)**				
A	70 (38.5)	13 (27.1)	57 (42.5)	0.883
B	32 (17.6)	13 (27.1)	19 (14.2)	0.598
0	66 (36.3)	13 (27.1)	53 (39.6)	0.116
AB	14 (7.7)	9 (18.8)	5 (3.7)	0.105
**Clinical characteristics, n (%)**				
Smoking	73 (40.1)	14 (29.2)	59 (44)	0.277
Trauma/surgery	37 (20.3)	3 (6.3)	34 (25.4)	0.199
Unprovoked VTE	92 (50.6)	36 (75)	56 (41.8)	0.020
DVT with PE	55 (30.2)	11 (22.9)	44 (32.8)	0.422
Family history of VTE	27 (14.8)	8 (16.7)	19 (14.2)	0.883
Proximal DVT	137 (75.3)	44 (91.7)	93 (69.4)	0.132
**Baseline laboratory parameters**				
INR	0.99 (0.91–1.05)	1.0 (0.96–1.1)	0.98 (0.89–1.1)	0.577
D-dimer, ng/mL	211 (156–270)	273 (191–296)	216 (146–254)	0.309
Fibrinogen, g/L	3.00 (2.51–3.93)	2.98 (2.56–4.08)	3.02 (2.45–3.87)	0.917
Creatinine, µmol/L	71.4 ± 14.1	73.5 ± 16.2	70.6 ± 13.2	0.577
Glucose, mmol/L	4.9 (4.5–5.3)	5.2 (4.8–5.6)	4.8 (4.5–5.1)	0.006
TC, mmol/L	5.08 ± 1.04	5.05 ± 0.93	5.10 ± 1.08	0.917
TG, mmol/L	1.31 ± 0.65	1.40 ± 0.65	1.29 ± 0.66	0.212
CRP, mg/L	1.73 (1.02–2.43)	2.17 (1.58–3.89)	1.44 (0.98–2.31)	0.076
IL-6, pg/mL	3.56 (2.76–4.04)	3.73 (3.03–4.00)	3.47 (2.67–4.06)	0.240
IL-10, pg/mL	6.45 (5.60–7.60)	6.50 (5.85–7.95)	6.40 (5.50–7.60)	0.418
HRG, µg/mL	68 (58–75)	76.5 (67.5–82)	64.5 (57–70)	< 0.001
Factor VIII, %	123 (102–142)	120 (103–139)	124 (101–143)	0.883
tPA Ag, ng/mL	9.2 (6.71–11.25)	7.62 (6.35–10.73)	9.60 (6.80–11.43)	0.577
PAI-1 Ag, ng/mL	12.28 (8.73–18.60)	10.89 (8.46–17.15)	12.90 (8.74–19.40)	0.426
TAFI activity, µg/mL	25.71 ± 7.06	32.01 ± 6.23	23.46 ± 5.88	< 0.001
Plasminogen, %	108.8 ± 15.2	106.6 ± 12.7	109.6 ± 15.9	0.445
α-antiplasmin, %	105 (96–116)	101 (92–113)	107 (98–117)	0.243
Peak thrombin, nM	231 (199–300)	219 (193–291)	235 (200–304)	0.577
**Fibrin clot formation and features**				
Plasma clot permeability, 10^−9^cm^2^	7.4 (6.5–8.4)	6.6 (6.0–7.6)	7.8 (6.9–8.8)	0.001
Clot lysis time, min	88 (73–101)	100 (88–109)	82 (70–97)	< 0.001
Lag time, sec	40 (37–45)	38 (35–41)	42 (38–47)	0.011
Maximum absorbance	0.81 (0.75–0.87)	0.85 (0.81–0.89)	0.80 (0.74–0.86)	0.006
Maximum levels of D‐dimer released from clots, mg/L	3.93 (3.59–4.35)	3.73 (3.60–4.30)	4.08 (3.57–4.39)	0.883
Maximum rate of increase in D-dimer levels released from clots, mg/L/min	0.071 (0.067–0.078)	0.0069 (0.063–0.073)	0.072 (0.068–0.079)	0.207
**Genetic polymorphisms, n (%)**				
Factor V Leiden	23 (12.6)	5 (10.4)	18 (13.4)	0.561
Prothrombin 20210A	7 (3.9)	0 (0)	7 (5.2)	0.557
Factor XIII Val34Leu	83 (45.6)	25 (52.1)	58 (43.3)	0.418
α-fibrinogen Thr312Ala	83 (45.6)	22 (45.8)	61 (45.5)	0.616

*PTS* post-thrombotic syndrome; *VTE* venous thromboembolism; *DVT* deep vein thrombosis; *PE* pulmonary embolism; *IL* interleukin; *INR* international normalized ratio; *TC* total cholesterol; *TG* total triglycerides; *CRP* C-reactive protein; *HRG* histidine-rich glycoprotein; *tPA* tissue plasminogen activator; *PAI-1* plasminogen activator inhibitor-1; *TAFI* thrombin activatable fibrinolysis inhibitor.

Median Villalta score was 16 (range 6–27).

Median HRG level in the total cohort of DVT patients was 68 (IQR 58–75) µg/mL. The HRG concentration for the whole cohort correlated with age (*r* = 0.24, *p* < 0.001) and glucose (*r* = 0.16, *p* = 0.03) and tended to be positively associated with BMI (*p* = 0.06) and CRP (*p* = 0.07). Of note, HRG levels were associated with the blood types (Supplemental Fig. 1).

**Figure 2:**
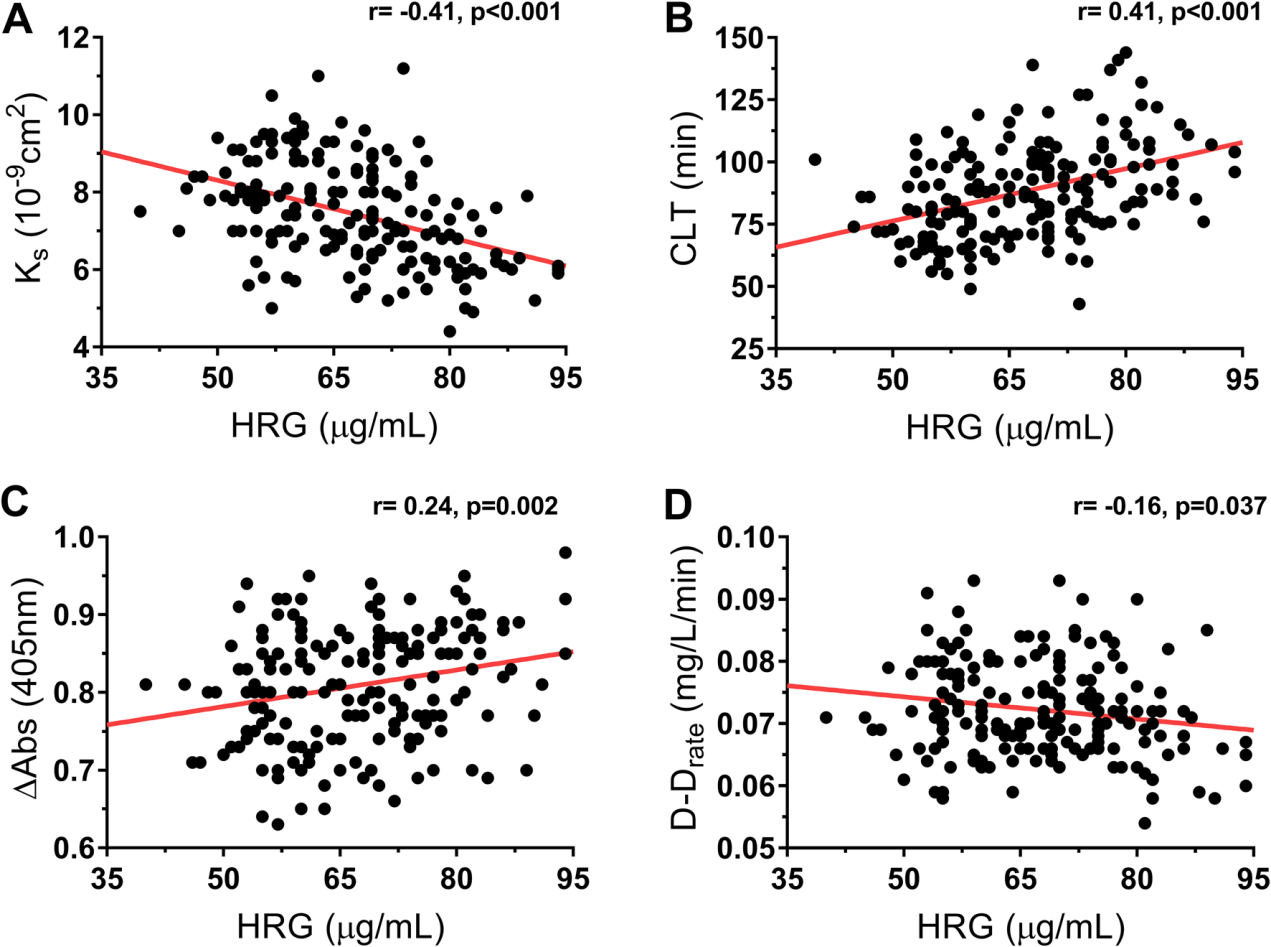
Correlations of the histidine-rich glycoprotein (HRG) with four variables measured 3 months after the index event in patients who developed post-thrombotic syndrome (PTS) (n = 48). Associations of HRG with (**A**) clot permeation coefficient (plasma clot permeability), (**B**) clot lysis time, (**C**) maximum absorbance of fibrin gel at 405 nm determined with turbidimetry (maximum absorbance), and (**D**) maximum D-dimer levels in the lysis assay.

Analysis of fibrin clot properties showed that baseline HRG levels correlated with plasma clot permeability (*r* = − 0.41, *p* < 0.001), maximum absorbance (*r* = 0.24, *p* = 0.002), and two fibrinolysis measures, i.e. clot lysis time (*r* = 0.41, *p* < 0.001) and maximum rate of increase in D-dimer levels released from clots (*r* = − 0.16, *p* = 0.04; Fig. 2). Moreover, baseline HRG levels showed a positive association with TAFI activity (*r* = 0.25, *p* = 0.001), but not with other fibrinolytic proteins or FVIII.

**Figure 3:**
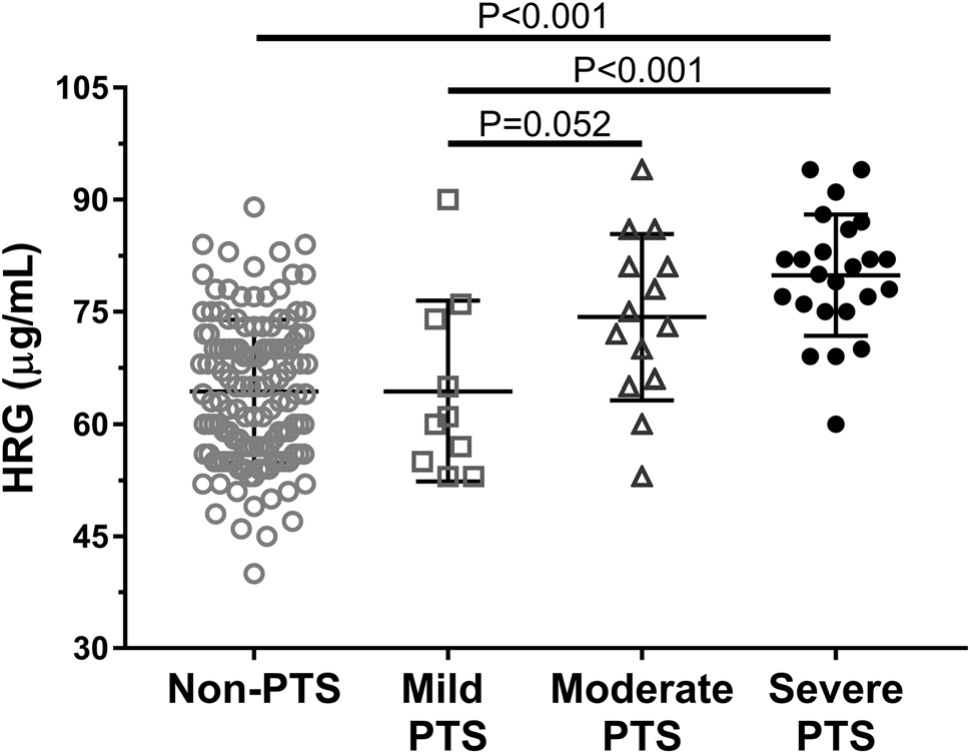
Distribution of baseline histidine-rich glycoprotein (HRG) levels among patients developed post-thrombotic syndrome (PTS). Severity of PTS was categorized according to the Villalta score: mild (5–9 points, n = 10), moderate (10–14 points, n = 14), and severe disease (≥ 15 points, n = 24). Data are presented as median, interquartile range, and maximum/minimum values.

As many as 48 patients were diagnosed with PTS after 12–14 months of observation, including 10 patients (20.8%) with mild PTS, 14 with moderate (29.2%), and 24 patients (50%) with Villalta score of ≥ 15 representing severe PTS. Patients with PTS were older, more frequently obese and had higher plasma glucose (Table 1). Baseline HRG was 18.6% higher in patients who developed PTS as compared to those who did not (*p* < 0.001). In patients with PTS, baseline HRG correlated with the Villalta score (*r* = 0.45, *p* = 0.001), but not with CRP. Severe PTS was associated with 24% and 8% higher HRG levels as compared with mild and moderate forms of the disease, respectively (Fig. 3). There was no difference in the prevalence of any of the 4 genetic polymorphisms tested (Table 1).

PTS patients displayed more prothrombotic clot features, including shorter lag phase and higher maximum absorbance during plasma clot formation, together with lower plasma clot permeability and impaired fibrinolysis as evidenced by prolonged clot lysis time (Table 1). Analysis of fibrinolytic proteins showed solely elevated TAFI activity in the PTS group (Table 1).

After 24 months in 131 patients (71.6%) HRG assessment was repeated (median 68, IQR 57–76 µg/mL). HRG levels remained unchanged compared to the baseline values (*p* = 0.39).

At the 24-month follow-up 48 patients (26.4%) had PTS. After 24 months patients with PTS compared with those without PTS had 18.5% higher levels of HRG (median 74.6, IQR 66–80 µg/mL vs. 65.6, IQR 60–72 µg/mL, *p* < 0.001). In the multivariate analysis adjusted for age, fibrinogen, and glucose we showed that recurrent VTE along with plasma HRG and TAFI activity were associated with PTS at 24 months (Table 2).

**Table 2:** Predictors of post-thrombotic syndrome (PTS) during 2 years’ follow-up.

Variable	OR per	Univariate		Multivariate*	
		OR (95% CI)	*p-*value	OR (95% CI)	*p-*value
Age	1 year	1.04 (1.01–1.07)	0.005	1.01 (0.98–1.04)	0.491
BMI	1 kg/m^2^	1.17 (1.06–1.30)	0.002	1.07 (0.95–1.22)	0.252
Unprovoked VTE	No/yes	2.57 (1.34–5.07)	0.005		
Proximal DVT	No/yes	2.26 (1.03–5.37)	0.041		
VTE recurrence	No/yes	10.25 (4.80–22.75)	< 0.001	2.68 (1.10–6.54)	0.031
Glucose	1 mmol/L	1.91 (1.19–3.17)	0.007		
HRG	1 µg/mL	1.08 (1.05–1.12)	< 0.001	1.06 (1.02–1.10)	0.004
CRP	1 mg/L	1.27 (1.07–1.54)	0.006		
TAFI activity	1 µg/mL	1.18 (1.11–1.25)	< 0.001	1.12 (1.05–1.20)	< 0.001
TAFI antigen	1%	1.04 (1.02–1.06)	0.001		
K_s_	1 × 10^−9^cm^2^	0.61 (0.45–0.80)	< 0.001		
CLT	1 min	1.04 (1.02–1.06)	< 0.001		

For abbreviations, please see Table 1. Multivariate model where variable selection was performed using least absolute shrinkage and selection operator (LASSO) algorithm and adjusted for age, fibrinogen and glucose.

At final assessment, after a median follow-up of 53 (IQR 47–76) months, PTS was diagnosed in 56 (32.6%), including 20 patients with mild PTS (35.7%), 12 with moderate (21.4%), and 24 patients (42.9%) with severe PTS. The baseline HRG levels were similar in patients who developed PTS after about 24 months and in those who developed PTS within the whole follow-up period (median, 76.5, IQR 67.5–82 µg/mL vs. 77, IQR 68–83 µg/mL, *p* = 0.89).

At final assessment, VTE recurred in 45 patients (24.7%) including 30 patients (53.6%) with PTS. Patients with PTS had higher rates of VTE recurrence than those who did not have PTS (*p* < 0.001). VTE recurrence occurred earlier in patients with PTS (median 11, IQR 7–39 months) than those without PTS (median 29, IQR 27–35 months; *p* < 0.001). Of note, HRG at baseline was 13.6% higher in patients with PTS who experienced VTE recurrence during follow-up than non-PTS cases who developed VTE recurrence. Multivariate analysis showed that baseline plasma clot permeability, TAFI activity, and CRP, but not HRG levels were significant predictors of recurrent VTE after adjustment for age, BMI and fibrinogen (Table 3).

**Table 3:** Predictors of recurrent venous thromboembolism.

Variable	OR per	Univariate		Multivariate*	
		OR (95% CI)	*p-*value	OR (95% CI)	*p-*value
BMI	1 kg/m^2^	1.15 (1.04–1.27)	0.005	1.05 (0.93–1.19)	0.40
PTS	No/yes	10.25 (4.80–22.75)	< 0.001	–	–
Smoking	No/yes	0.48 (0.22–0.98)	0.043	–	–
Trauma	No/yes	0.22 (0.05–0.64)	0.004	–	–
Proximal DVT	No/yes	3.51 (1.40–10.71)	0.006	–	–
Fibrinogen	1 g/L	1.38 (0.97–1.96)	0.072	0.92 (0.59–1.43)	0.72
HRG	1 µg/mL	1.07 (1.04–1.27)	0.004	–	–
CRP	1 mg/L	1.39 (1.16–1.72)	< 0.001	1.27 (1.00–1.61)	0.047
TAFI activity	1 µg/mL	1.14 (1.08–1.21)	< 0.001	1.08 (1.01–1.15)	0.023
Plasma clot permeability	1 × 10^−9^cm^2^	0.36 (0.24–0.52)	< 0.001	0.43 (0.27–0.66)	< 0.001
Maximum absorbance (405 nm)	0.01	1.16 (1.10–1.24)	< 0.001	–	–
Clot lysis time	1 min	1.04 (1.02–1.07)	< 0.001	1.01 (0.98–1.04)	0.47

For abbreviations, please see Table 1. Multivariate model where variable selection was performed using least absolute shrinkage and selection operator (LASSO) algorithm and adjusted for body mass index (BMI) and fibrinogen.

## Discussion

This study is the first to show that elevated plasma HRG levels measured at 3 months since first-ever DVT are associated with the development of PTS. Moreover, we provided evidence that unfavorable effects of elevated HRG are at least in part associated with alterations to fibrin clot structure and impaired fibrinolysis observed in patients who developed PTS during follow-up. In contrast, elevated HRG did not associate with VTE recurrence. In light of inconsistent data on the role of HRG in human thrombosis^21–24^, our findings provide new insights in this regard suggesting a negligible impact of HRG on the risk of recurrent VTE in real-life patients. The current study suggests that HRG could be a yet unknown factor of the risk of developing PTS and might help identify DVT patients at increased risk of this common complication. Mechanisms underlying this association are likely multiple with a significant contribution of the prothrombotic fibrin clot phenotype enhanced by elevated HRG. It has been previously shown that HRG interacts with fibrinogen^18,28^. Furthermore, not only does HRG have a high affinity for fibrinogen, but it is also incorporated into fibrin clots generated from human plasma in vitro^20,28^. It is known that the presence of HRG in fibrin clots significantly affects the structure of the clot by causing the formation of thinner fibrils^18^, which results in denser networks that are usually more resistant to lysis^11^. Since fibrin is the main component of all venous thrombi^29,30^, elevated circulating HRG levels likely lead to augmented incorporation of this protein into fibrin networks and the subsequent disturbed degradation of fibrin and possible harmful local actions. Our findings suggest that HRG may contribute to the development of PTS via prothrombotic alterations to fibrin structure and function. Moreover, elevated HRG along with recurrent VTE and increased TAFI activity, reported previously^11^, independently predicted PTS. This mechanism is further supported by the fact that HRG levels remained unaltered after 2 years since the first measurement, suggesting the elevation of HRG is a persistent characteristic in a proportion of VTE patients. Our observations regarding hypofibrinolysis, reflected by prolonged clot lysis time are in line with the studies performed on mice with HRG deficiency, in which accelerated fibrinolysis has been reported^19,31^. Taken together, it might be speculated that elevated HRG has a prothrombotic impact on fibrin clot structure and function, including impaired lysis.

Given accumulating data on the key role of inflammation in PTS, the current study is in line with previous reports^13,32^ by showing that patients at risk of PTS tended to have elevated CRP levels compared with those free of this adverse event, without any differences in concentrations of IL-6 or IL-10. Regarding a potential effect of inflammation on HRG levels, it is worth mentioning that decreased HRG levels have been suggested as a novel biomarker for sepsis^13^. In such cases, the values of CRP during systemic inflammatory response syndrome and sepsis tend to be much higher—typically greater than 150 mg/L^33^—than in VTE. For this reason, this inverse relationship between HRG and CRP in systemic inflammatory response syndrome and sepsis cannot be expected in typical DVT patients. There was no correlation between HRG and CRP in the present study. Moreover, it has been shown that HRG is distributed systemically as an unbound form, bound with plasminogen, and bound with platelets, phagocytes or other cells^34^, which are involved in the inflammatory response. Our data suggests that in PTS patients, in which inflammatory markers were relatively low and did not differ from non-PTS patients elevated HRG levels are associated rather with impaired fibrinolysis than inflammation. The issue of the association between the HRG levels and inflammation is worth investigating in future studies.

It should be highlighted that the current study showed a relatively high percentage of severe PTS patients and recurrent events among them. This unexpected finding might be related to several issues, such as the lack of anticoagulation clinics in Poland, which are known to offer the best long-term care for patients with PTS together with education^35^. Most patients referred to our clinic had more severe clinical course of DVT, they had mostly proximal DVT (75%). Further, even if patients declared that they implemented the compression therapy we were not able to assess the compliance during log-term follow-up. Since the most available studies on PTS were performed in high income countries with less than 10% patients with severe PTS, the current study could be also important by showing a large group of such patients, which highlights the need or improved care provided in DVT patients to minimize the risk of severe PTS. Thus, additional value of this report is the analysis of relatively high number of patients with the Villalta score ≥ 15, which are usually underpowered in studies regarding PTS.

Our study has several limitations. The number of patients with PTS was limited with the prevalence of the syndrome similar to that reported in other studies^1^. Any subgroup analysis within the PTS group should be interpreted with caution. In addition, this study excluded patients with known thrombophilic states, the elderly, and significant comorbidities such as cancer or diabetes, as it has been shown that these conditions unfavorably alter plasma fibrin properties regardless of VTE^36^. Therefore, our findings likely cannot be extrapolated to such populations. At this point, it is too early to suggest that HRG may be used in clinical practise as a predictor of PTS. In vitro studies would be needed to better ascertain the function of HRG in patients following DVT, explore potential mechanisms linking this protein with PTS, and finally larger scale studies to determine reference ranges for HRG. We did not determine the HRG Pro186 allelic variant, which has been reported more frequently in thrombosis recently^37^. No data linking this variant with PTS has been published.

In conclusion, we reported here an original finding suggesting that elevated HRG is associated with the development of PTS, which might be in part associated with impaired clot lysis. Further large cohort studies are needed to corroborate these findings and mechanistic investigations should look into how HRG may contribute to long-term complications of VTE.

## Supplementary information

Supplementary file1
