# Supplementary material for: Increased levels of histidine-rich glycoprotein are associated with the development of post-thrombotic syndrome
Source: Sci Rep. 2020 Sep 2;10:14419. doi: 10.1038/s41598-020-71437-5 (PMC7468120; doi:10.1038/s41598-020-71437-5)

**Increased Levels of Histidine-Rich Glycoprotein are Associated with the Development of Post-Thrombotic Syndrome**

*Jakub Siudut^1,2^, *Joanna Natorska^1,2^, Maksim Son^3^, Krzysztof Plens^4^, and Anetta Undas^1,2^

^1^Krakow Centre for Medical Research and Technologies, John Paul II Hospital; Krakow, Poland

^2^Institute of Cardiology, Jagiellonian University Medical College; Krakow, Poland

^3^Department of Clinical Neurological Sciences, University of Western Ontario; London, Canada

^4^KCRI, Krakow, Poland

*equally contributed authors

Supplemental Figure 1. Blood type associations with HRG levels.


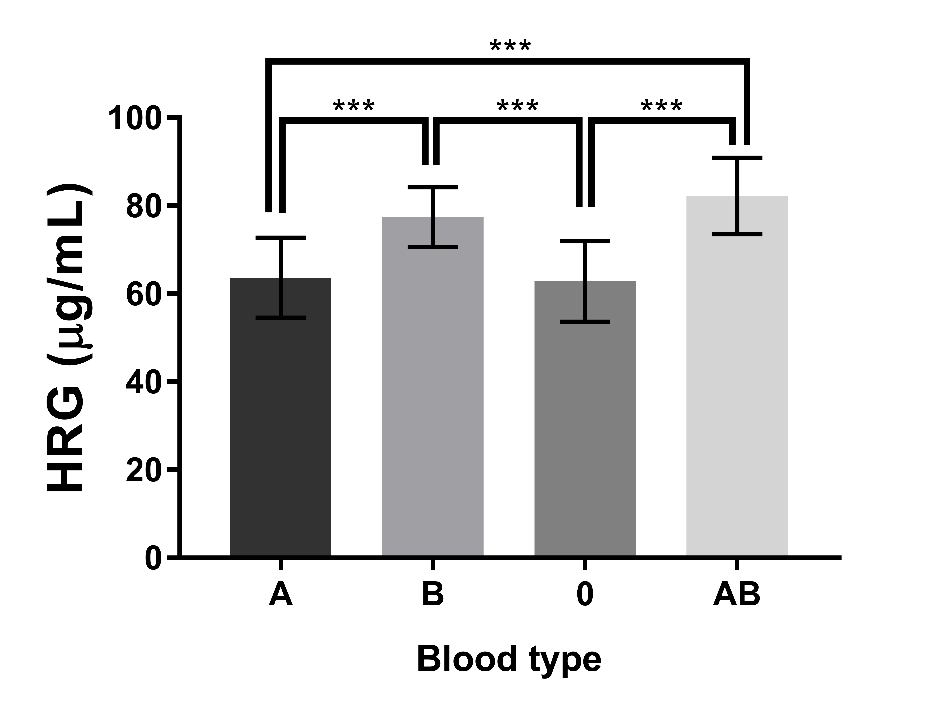

Supplement: Supplementary file 1 — Supplementary file1 [file 41598_2020_71437_MOESM1_ESM.docx]
